# Supplementary material for: Genetic mapping of craniofacial traits in the Mexican tetra reveals loci associated with bite differences between cave and surface fish
Source: BMC Ecol Evol. 2023 Aug 25;23:41. doi: 10.1186/s12862-023-02149-3 (PMC10463419; doi:10.1186/s12862-023-02149-3)
Supplement: Supplementary file 1 — Additional file 1: Supplemental Table 1. Gape angle statistics. [file 12862_2023_2149_MOESM1_ESM.pdf]

Supplemental Table 1. Gape angle statistics

	**Post-hoc Tukey's HSD**				**Two-Tailed T-test**	
Population	*diff*	*lwr*	*upr*	*p adj*	*Test Statistic*	*p-value*
Pachón - F2 OB	19.106292	7.156578	31.05601	0.000855	4.77045	0.0003
Surface - F2 OB	-10.52392	-22.47363	1.425797	0.099398	2.3684	0.03279
F2 UB - F2 OB	13.804708	1.854995	25.75442	0.018873	2.43862	0.02866
Surface - Pachón	-29.63021	-41.57992	-17.6805	1.4E-06	11.83765	1.11E-08
F2 UB - Pachón	-5.301583	-17.2513	6.64813	0.624994	1.23039	0.23882
F2 UB - Surface	24.328625	12.37891	36.27834	3.43E-05	5.15538	0.00015
