# Supplementary material for: Genetic mapping of craniofacial traits in the Mexican tetra reveals loci associated with bite differences between cave and surface fish
Source: BMC Ecol Evol. 2023 Aug 25;23:41. doi: 10.1186/s12862-023-02149-3 (PMC10463419; doi:10.1186/s12862-023-02149-3)
Supplement: Supplementary file 2 — Additional file 2: Supplemental Table 2. Tooth mark statistics. [file 12862_2023_2149_MOESM2_ESM.pdf]

Supplemental Table 2. Tooth mark statistics

	Post-hoc Tukey's HSD				Two-Tailed T-test	
Population	diff	lwr	upr	p adj	Test Statistic	p-value
Pachón - F2 OB	4.398473	1.970668	6.826278	0.000475	4.20103	0.00299
Surface - F2 OB	1.058394	-1.369411	3.486199	0.607415	1.32705	0.22111
F2 UB - F2 OB	2.986504	0.558699	5.414309	0.013599	3.32031	0.01054
Surface - Pachón	-3.340079	-5.767884	-0.912274	0.005835	4.2043	0.00298
F2 UB - Pachón	-1.41197	-3.839775	1.015835	0.373475	1.57461	0.15399
F2 UB - Surface	1.92811	-0.499695	4.355915	0.146412	3.28754	0.01106
