# Supplementary material for: Genetic mapping of craniofacial traits in the Mexican tetra reveals loci associated with bite differences between cave and surface fish
Source: BMC Ecol Evol. 2023 Aug 25;23:41. doi: 10.1186/s12862-023-02149-3 (PMC10463419; doi:10.1186/s12862-023-02149-3)
Supplement: Supplementary file 3 — Additional file 3: Supplemental Table 3. Genetic marker alignment to the Pachón and surface fish genomes. [file 12862_2023_2149_MOESM3_ESM.pdf]

Supplemental Table 3. Genetic marker alignment to the Pachón and surface fish genomes

Genetic Marker	LOD	Linkage Group	Position (cM)	Pachón (AstMex102) Scaffold	Position (bp)	Surface (Asty 2.0) Genome Position
r51027	3.162	1	95.02952	KB871706	75289	7:3972216-3990923
r22314	4.359	1	92.16596	KB871620	110904	7:8403689-8414374
r22424	4.359	1	92.16596	KB871620	395628	7:8541483-8547540
r22459	4.359	1	92.16596	KB871620	449201	7:8608771-8614831
r22285	4.549	1	91.48504	KB871620	1018988	7:9195093-9270150
r187365	4.549	1	91.48504	KB882095	157813	7:9290842-9429546
r52534	4.708	1	90.68322	KB871713	145163	7:10210392-10236794
r81835	4.032	1	87.49341	KB871833	888903	7:12687159-12699817
r80566	2.67	1	86.36256	KB871833	1859884	7:12772597-12800458
