# Supplementary figures and images for: Genetic mapping of craniofacial traits in the Mexican tetra reveals loci associated with bite differences between cave and surface fish
Source: BMC Ecol Evol. 2023 Aug 25;23:41. doi: 10.1186/s12862-023-02149-3 (PMC10463419; doi:10.1186/s12862-023-02149-3)

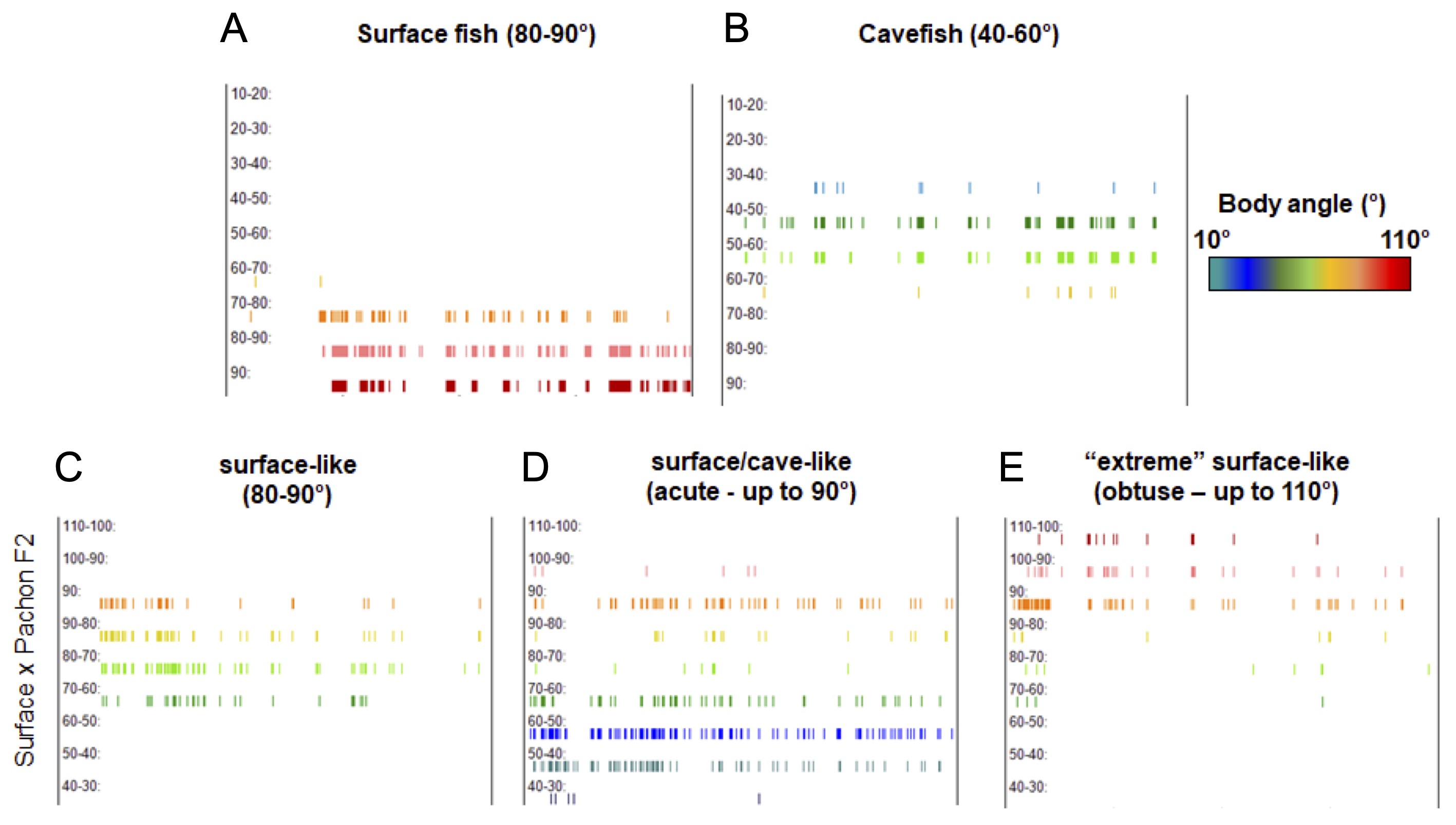

Supplement: Supplementary file 6 — Additional file 6: Supplemental Figure 1. Ethograms illustrate feeding posture differences between surface, cavefish and hybrids. Consistent with findings from Kowalko et al. [23], we determined that surface fish have a near perpendicular feeding posture with an average angle between 80°-90° (A) and cavefish feed at a lower angle of 40°-60° (B). Surface x Pachón F2 hybrids demonstrated three feeding posture categories: surface-like F2 hybrids with an average feeding angle between 80°-90° (C), a mix of surface- and cave-like feeding postures with angles ranging from 40°-90° (D), and an extreme obtuse posture with angles up to 110° (E). [file 12862_2023_2149_MOESM6_ESM.jpg]
